# A novel Nutrient Rich Food (NRFa11.3) score uses flavonoids and carotenoids to identify antioxidant-rich spices, herbs, vegetables, and fruit

**DOI:** 10.3389/fnut.2024.1386328

**Published:** 2024-04-18

**Authors:** Adam Drewnowski

**Affiliations:** Center for Public Health Nutrition, University of Washington, Seattle, WA, United States

**Keywords:** nutrient profiling, Nutrient Rich Food (NRF) index, antioxidants, carotenoids, flavonoids, herbs, spices, SR-28

## Abstract

**Introduction:**

Nutrient profiling (NP) models designed to evaluate the healthfulness of plant-based foods ought to incorporate bioactive phytochemicals. Herbs and spices are one food group of current interest.

**Methods:**

Two new versions of the well-established Nutrient Rich Food (NRF) index were applied to spices, herbs, vegetables, fruit, and other plant-based foods. Analyses used the US Department of Agriculture (USDA) SR-28 nutrient composition database merged with the USDA Expanded Flavonoid database 3.3. The NRF4.3 model was based on protein, fiber, potassium, and vitamin C. The NRFa11.3 model was based on micronutrients with reported antioxidant activity (vitamin C, vitamin E, selenium, copper, and zinc), carotenoids (alpha and beta carotene, beta-cryptoxanthin, lycopene, lutein/zeaxantin) and flavonoids. Saturated fat, added sugar, and sodium were nutrients to limit. The NRF algorithm was based on sums of percent daily values (%DVs) capped at 100%.

**Results:**

The NRF4.3 model awarded high scores to herbs and spices, cocoa powder, and nuts, but did not discriminate well among vegetables and fruit. The NRFa11.3 model performed better. Green leafy, red orange and cruciferous vegetables had the highest carotenoid content. Highest in flavonoids were cocoa powder, herbs and spices, and berries. Highest combined NRFa11.3 values were observed for herbs and spices, green leafy vegetables, cocoa, nuts, and red-orange and cruciferous vegetables.

**Discussion:**

Fresh and dry herbs and spices, often ignored by NP models, were particularly nutrient-rich and may provide non-negligible amounts of key phytonutrients to the human diet.

## 1 Introduction

Nutrient profiling (NP) models are quantitative methods intended to capture nutrient density of foods (1–3). The definition of nutrient density often depends on population health needs (4). In countries where obesity and diabetes are major concerns, NP models typically penalize foods that are high in calories, saturated fat, sugars, and sodium (5). In countries where micronutrient deficiencies still pose a risk, NP models might also include those priority micronutrients that are missing from the population diet, notably iron, zinc, calcium, iodine, folate, and vitamin A (6). Dietary inadequacies are not limited to lower-income countries. Vulnerable populations in high-income countries can have diets that are inadequate in vitamins, minerals, and functional ingredients from plants (7).

Recognizing the health benefits of vegetables and fruit (8), the Dietary Guidelines for Americans (DGA) have long promoted their inclusion in healthy sustainable diets (9). Global dietary guidelines have done the same (10). Yet the consumption of vegetables and fruit in the US remains well below recommended values (9).

Missing from the US population diet are not just potassium and fiber, recognized as nutrients of concern (9) but also carotenoids, flavonoids, and trace elements. Many of these compounds are also found in herbs and spices (11). Dried or dehydrated culinary herbs are natural plant products that have been dried without any additional processing. The US Food and Drug Administration (FDA) defines a spice as any aromatic vegetable substance in the whole, broken, or ground form whose significant function is in providing seasoning rather than any nutritional value (12). Perhaps for that reason, the nutritional value of spices has been relatively understudied, and no formal investigation of nutrient density of herbs and spices has been attempted so far.

Capturing nutrient density of foods is always a challenge. Many NP models rely on fiber, sugar, and sodium to discriminate among different vegetables and fruit. The French NP model Nutri-Score awards points for the percent by weight of *any* fruit, vegetable, or nut in a given food, failing to make any discrimination at all (13). An NP model that could better capture the relative healthfulness of herbs and spices, vegetables, and fruit (14) would be a contribution to the literature and might help in the implementation of dietary guidance. A focus on herbs and spices would be useful; this understudied category is often viewed as an alternative to sodium but can also provide important nutrients and dietary components.

The present narrowly defined objective was to devise a category-specific NP model (14) that was based on the classic NRF algorithm and featured both nutrients and functional ingredients. One important aspect of the Nutrient Rich Foods (NRF) framework is its flexibility allowing for the substitution of one nutrient for another (2, 3). The elements new to nutrient profiling were carotenoids, flavonoids, and selected vitamins and trace minerals as described in detail below. Nutrient composition data came from the USDA SR-28 database (15) merged with the USDA data on flavonoid content of selected foods, release 3.3 (16).

## 2 Methods

### 2.1 The USDA SR-28 database

The new NRF models were developed using the open-access USDA National Nutrient Database for Standard Reference, Release 28 (now SR Legacy), that is available on FoodData Central (15). The SR-28 database lists total amounts of nutrients present in the edible portion of the food, including any nutrients added in processing. The nutrient content values are for fruits and vegetables that are fresh, frozen, or canned, sometimes with the addition of other ingredients such as sugar syrup in canned fruit. Herbs and spices can be dried or fresh. NDB (National Data Base) first digit codes were used to identify fruits, vegetables, beans, nuts, grains, and herbs and spices.

Based on the USDA classification used in the What We Eat in America (WWEIA) studies (17), the fruit category was subdivided into citrus fruit, citrus juice, other fruit (raw or cooked), melons, dried fruit, berries, and olives. Fruit jams, jellies and preserves other than cranberry were excluded. Vegetables were subdivided into leafy green vegetables, cruciferous vegetables, red/orange vegetables (and products), and other vegetables. Beans were chickpeas, lima beans, lentils and soy. Additional categories were nuts, cocoa powder, chocolate, coffee, and tea, and selected whole grains.

The herbs and spices category included dried herbs and spices, along with some fresh herbs. Items for which nutrient composition data were available (albeit with some missing values) included allspice, anise, basil, bay leaves, caraway seed, cardamon, celery seed, chervil, chili powder, cinnamon, cloves, coriander, cumin seed, dill seed, fennel seed, fenugreek, ginger, mace, marjoram, mustard, nutmeg, oregano, paprika, parsley, pepper (black, red, or white), rosemary, saffron, sage, tarragon, turmeric, and thyme. The SR-28 spices category also listed vinegars; those were removed.

The SR-28 provides energy and nutrient content for 65 nutrients per 100 g of food for 8,789 foods from all food groups. In addition to protein, fiber and vitamins and minerals, the SR-28 provides data on individual carotenoids (β-carotene, α-carotene, β-cryptoxanthin, lycopene, and lutein+ zeaxanthin). Most analytical systems do not separate lutein and zeaxanthin, so these carotenoids are typically combined. The SR-28 also lists values for vitamin C, vitamin E, selenium, copper, and zinc. As might be expected, there were some missing values.

### 2.2 The USDA expanded flavonoid database

The USDA database for the flavonoid content of selected foods, release 3.3 (16) contains analytical values for 25 flavonoid compounds for 512 food items. The unit of measure is mg/100 g edible portion on fresh weight basis. Subclasses of flavonoids were flavonols, flavones, flavan-3-ols, flavanones, and anthocyanidins. Detailed documentation is provided online (16). The term “total flavonoids” refers to those flavonoids that were listed in the release 3.3 of the USDA database.

### 2.3 Merging SR-28 with the expanded flavonoid database

The SR-28 database and the much smaller USDA Expanded Flavonoid database version 3.3. were matched using NDB food identification codes. Some herbs and spices lacked flavonoid data and could not be matched, indicating a potential data need. Analyses that involved flavonoids were restricted to those items where the match was successful. A total of 335 items in multiple categories of interest were available for data analysis.

### 2.4 Adapting the NRF algorithm for plant-based products

As new datasets become available, the classic NRF algorithm structure can be maintained but some index nutrients can be easily swapped, added, or removed. Notably, the choice of index nutrients can vary depending what available data can best capture nutritional value of items of interest.

The NRF9.3 algorithm is based on the unweighted sum of percent Daily Values (%DV) for 9 nutrients to encourage (the NR9 sub-score) minus the sum of %DV for 3 nutrients to limit (LIM sub-score) (18). The original Nutrient Rich (NR9) sub-score was based on protein, fiber, vitamins A, C and E, calcium, iron, potassium, and magnesium (2, 3). Later versions replaced vitamin E with vitamin D. Those nutrients are either part of the FDA definition of « healthy » or have been identified by the DGA as shortfall nutrients under-consumed by the US population. Nutrients to limit were saturated fat, added sugars, and sodium.

The NRF algorithm uses the sum rather than the mean of %DVs. NRF algorithms based on subtraction (NR9 – LIM) yielded a better distribution of values than did those based on ratios (NR9/LIM). The final product was given the name NRF9.3. The %DVs were expressed per 100 kcal and were capped at 100%.

### 2.5 Two NRF scores: NRF4.3 and NRFa11.3

The NRF4.3 score was based on the sum of %DVs for 4 nutrients to encourage: protein, fiber, vitamin C and potassium (the NR4 sub score) minus the sum of %DVs for saturated fat, added sugar and sodium (LIM sub score). These 4 nutrients to encourage are normally listed on the Nutrition Facts Panel.

The NRFa11.3 was also based on the sum of %DV nutrients and components to encourage (NR11 sub score) minus the sum of %DV for nutrients to limit. The elements to encourage were vitamins and minerals, carotenoids, and flavonoids. Vitamins and minerals were zinc, copper, selenium, vitamin C, and vitamin E. The 5 carotenoids were alpha carotene, beta carotene, beta cryptoxanthin, lycopene, and lutein/zeaxanthin. Total flavonoids were the sum of flavonoid classes. In a departure from the original NRF9.3, %DVs for both models were calculated per 100 g rather than per 100 kcal.

### 2.6 Selection of nutrient standards

Nutrient standards are typically based on local reference dietary amounts. The U.S. Reference Daily Values (19, 20) that are published by the FDA and used on nutrition labels are summarized in Table 1. In a departure from the original model, the present approach was to convert nutrient amounts per 100 g to percent daily values (% DV) also per 100 g. As in the past, percent DVs were capped at 100% so that foods containing very large amounts of a single nutrient would not have a disproportionately high score.

**Table 1:** Reference daily values (RDV) for nutrients to encourage and nutrients to limit based on a 2,000-kcal diet.

Nutrient	RDV	Nutrient	RDV
Protein	50 g	Saturated fat	20 g
Fiber	28 g	Total sugar	90 g
Vit C	90 mg	Added sugar	50 g
Vit E	15 mg	Sodium	2,300 mg
Zinc	11 mg		
Copper	0.9 mg		
Selenium	75 mcg		
Potassium	3,500 mg		
Alpha carotene	3,600 mcg		
Beta carotene	1,800 mcg		
Beta cryptoxanthin	3,600 mcg		
Lycopene	3,600 mcg		
Lutein/zeaxanthin	3,600 mcg		
Total flavonoids*	250 mg		

*There are no official reference daily values for flavonoids at this time.

Reference daily values for carotenoids were based on USDA calculations of the relations between provitamin A carotenoids and retinol equivalents (RAE). No daily values are available for flavonoids at this time. The present preliminary standards were based on estimated median intake of total flavonoids at 250 mg/d that was based on prior estimates of population intakes of flavonoids in the Us based on NHANES and other data (21, 22); observed associations between flavonoid intakes and diet quality and (23) and flavonoid intakes of tea consumers versus non-consumers (24).

The three nutrients to limit in the LIM sub-score of the NRF9.3 model were saturated fat, added sugar and sodium (2, 3). Maximum recommended values (MRVs) for nutrients to limit were 20 g for saturated fat, 90 g for total sugar, 50 g for added sugar, and 2,300 mg for sodium, all based on a 2000 kcal/d diet (18). Most of the food items selected contained no (or minimal amounts) of saturated fat, added sugars, or sodium.

### 2.7 Plan of analysis

Differences in NR sub scores and NRF scores by food category were tested using one way ANOVA with *post hoc* Dunnett’s test, using herbs and spices as the control group that was compared to all other groups. Statistical significance determined at *p* value <0.05. Analyses used SPSS (Statistical Package for the Social Sciences v 16.0).

## 3 Results

### 3.1 Standard NRF4.3 score applied to spices, herbs, vegetables, and fruit

**Figure 1:**
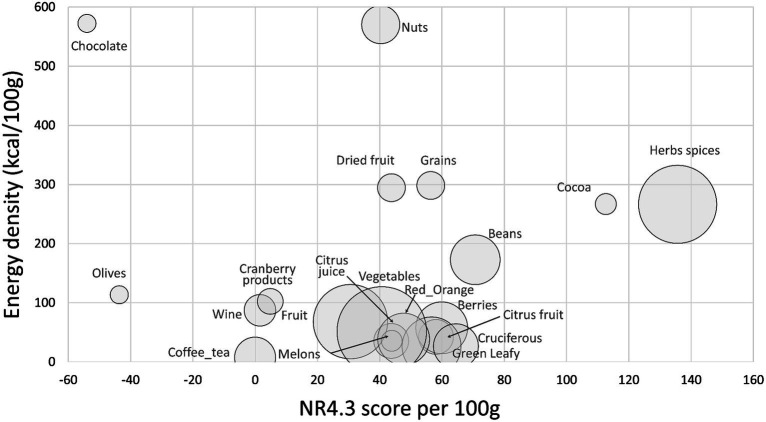
Scatterplot of NR4 scores plotted against energy density by food category. Size of bubble denotes number of items in category.

Figure 1 shows mean values for the standard NRF4.3 score (based on protein, fiber, potassium, vitamin C) by food category. Data are plotted against energy density (kcal/100 g) with the size of the bubble corresponding to the number of items in each category. On per 100 g basis, the highest NRF4.3 scores were obtained for herbs and spices, cocoa powder, beans, cruciferous vegetables, and berries. Nuts and dried fruit scored lower because of the fat and added sugar content. Olives and chocolate obtained negative scores (because of salt and sugar). As shown in Figure 1, the NRF4.3 score did not discriminate among fruits and vegetables particularly well. Berries scored higher than most other fruits largely because of high vitamin C content. Supplementary Table S1 shows means and standard errors (SEM) for NR4 sub score components protein, fiber, vitamin C, and potassium by food category. Highest in protein and fiber were herbs and spices, nuts, cocoa/chocolate, and beans. Herbs and spices and cocoa/chocolate were also highest in potassium. Berries were highest in vitamin C (acai berry was the highest), followed by cruciferous and red/orange vegetables, herbs and spices, leafy green vegetables, and citrus fruit.

Supplementary Table S2 shows energy density (in kcal/100 g), LIM and NRF4.3 values by food category. As expected, nuts had the highest energy density followed by chocolate, whole grains, dried fruit and herbs and spices (mostly dry). Highest LIM values were for chocolate (sugar), olives (salt), and nuts.

### 3.2 Carotenoid and flavonoid content of spices, herbs, vegetables, and fruit

**Figure 2:**
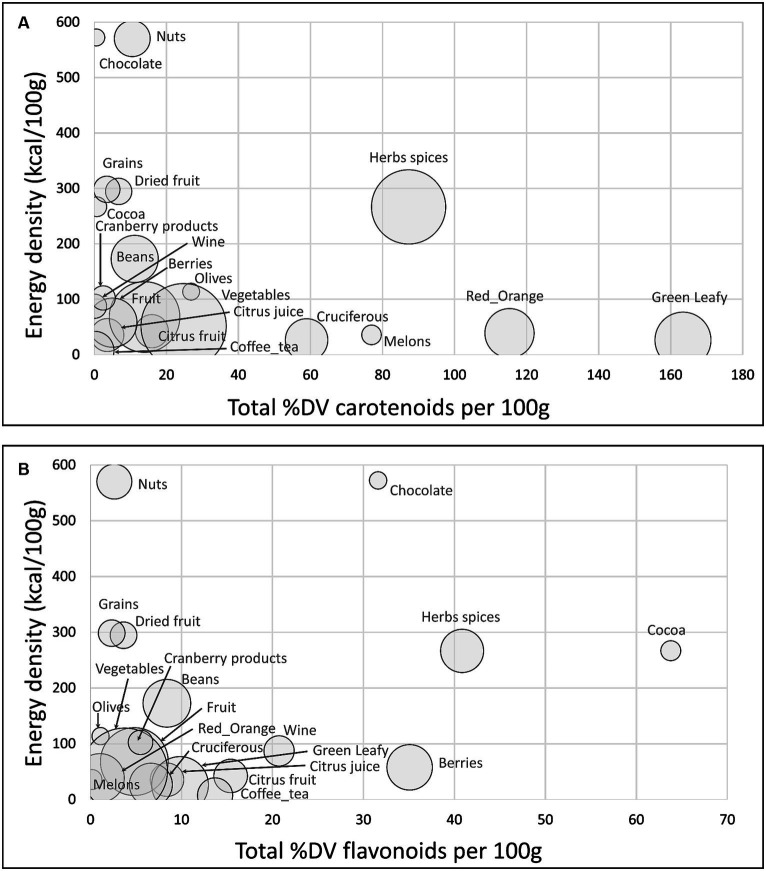
Scatterplots of total sum %DV of carotenoids **(A)**; total sum %DV of flavonoids **(B)** plotted against energy density by food category. Size of bubble denotes number of items in each category.

Figure 2A shows a scatterplot of %DV for carotenoids plotted against energy density. The size of the bubble denotes the number of items in each category. All calculations are per 100 g. Highest in carotenoid content were green leafy vegetables, red orange vegetables, herbs and spices, melons, and cruciferous vegetables.

Figure 2B shows a scatterplot of %DV for flavonoids plotted against energy density. Highest in flavonoids were cocoa powder and chocolate, herbs and spices, and berries. Flavonoid content in the USDA database was not available for all herbs and spices.

**Figure 3:**
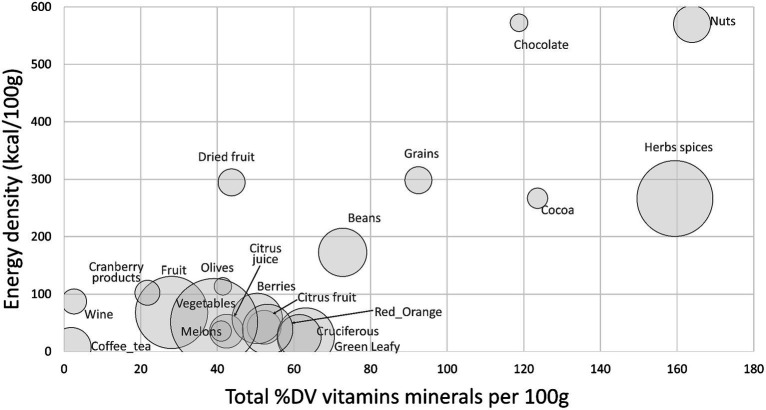
Scatterplot of total sum %DV of antioxidant vitamins and minerals (AVM) plotted against energy density by food category. Size of bubble denotes number of items in each category.

Figure 3 shows a scatterplot of %DV for antioxidant vitamins and minerals (AVM) plotted against energy density. The AVM were vitamin C, vitamin E, selenium, copper, and zinc. Highest in AVM (per 100 g) were nuts, herbs and spices, cocoa powder and chocolate, whole grains, and beans.

Data for sum %DV for total carotenoids, total flavonoids and AVM by food category are provided in Supplementary Table S3. Herbs and spices were consistently high in most carotenoids other than lycopene and were high in antioxidant vitamins and minerals.

### 3.3 A composite NRFa11.3 nutrient density score for spices, herbs, vegetables, and fruit

The final NRFa11.3 score was calculated as the sum of %DV for nutrients and other compounds to encourage (AVM, carotenoids and flavonoids) minus the sum of % DV for the nutrients to limit (saturated fat, added sugar, sodium). All individual %DV were capped at 100%.

**Figure 4:**
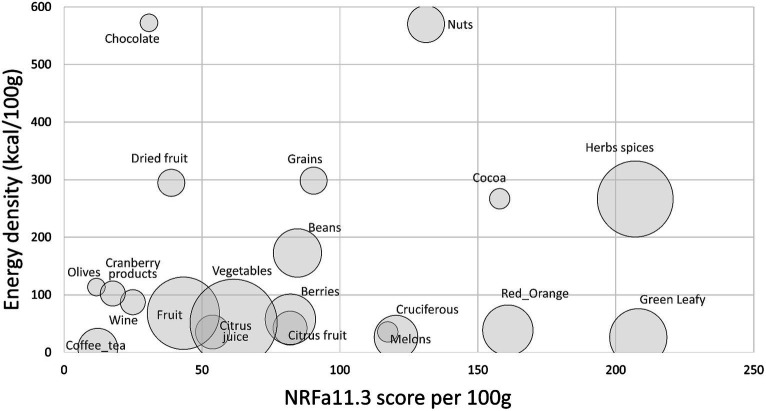
Scatterplot of aNRF11.3 values plotted against energy density by food category. Size of bubble denotes number of items in each category.

Figure 4 shows composite NRFa11.3 scores plotted against energy density by food category. Highest values were assigned to herbs and spices, green leafy vegetables, cocoa powders, nuts, and cruciferous vegetables. Berries were among the highest-scoring fruit. Data (means and SEM) and statistics are shown in Supplementary Table S4.

Top scoring items are shown in Table 2. Spices and herbs, fresh and dried, had some of the highest NRFa11.3 scores. Pepper, paprika and chili powder along with dried and some fresh herbs had the highest estimated content of nutrients and functional ingredients per 100 g. Leafy green vegetables also scored high, benefiting from high carotenoid content and low energy density. High scores were also obtained by red orange vegetables (pumpkin, squash, carrots), pulses, nuts, and fruit, especially melons and berries. Melons are high in carotenoids whereas berries score high because of high vitamin C and flavonoid content.

**Table 2:** Values of NRFa11.3 score for herbs and spices, leafy green and green vegetables, and other vegetables, nuts, and fruit.

Herbs and spices	NRFa11.3	Leafy green and green vegetables	NRFa11.3	Vegetables, nuts, fruit	NRFa11.3
Pepper, red or cayenne	542	Kale, raw	455	Pumpkin, raw	276
Paprika	530	Mustard greens, raw	338	Cowpeas, raw	264
Parsley, dried	517	Turnip greens, raw	331	Squash, Butternut, raw	262
Coriander leaf, dried	451	Cress, garden, raw	308	Guavas, raw	253
Parsley, fresh	421	Watercress, raw	284	Carrots, cooked, w/o salt	231
Chili powder	419	Chicory greens, raw	281	Carrots, baby, raw	230
Oregano, dried	404	Kale, cooked, w/o salt	280	Pistachio nuts, raw	229
Thyme, dried	400	Collards, raw	267	Peppers, sweet, red, raw	221
Marjoram, dried	357	Chard, Swiss, raw	266	Almonds	219
Peppers, hot chile, dried	349	Spinach, raw	266	Hazelnuts	218
Sage, ground	338	Mustard greens, cooked, w/o salt	260	Carrots, raw	218
Thyme, fresh	296	Taro leaves, raw	259	Tomato juice	203
Basil, dried	294	Spinach, frozen	251	Cabbage, red, raw	198
Celery seed	288	Radicchio, raw	250	Brazil nuts	194
Curry powder	287	Cress, garden, cooked w/o salt	244	Currants, black	187
Basil, fresh	277	Sweet potato leaves, raw	235	Tomato puree, cnd	178
Mustard seed, ground	275	Arugula, raw	228	Peas, green, raw	172
Cocoa powder	241	Sweet potato leaves, cooked	218	Beans, pinto, raw	167
Caraway seed	227	Broccoli Rabe, cooked	217	Papayas, raw	160
Coriander leaves, raw	227	Chives, raw	206	Cherries, tart, dried	153
Chervil, dried	218	Peppers, hot chile, green, raw	195	Raspberries, wild	153
Cumin seed	216	Peppers, jalapeno raw	193	Beans, white, raw	151
Saffron	210	Brussels sprouts, raw	184	Melons, Cantaloupe, raw	148
Coriander seed	194	Broccoli, raw	179	Elderberries, raw	148
Baking chocolate	183	Lettuce, Cos or Romaine	178	Chokeberries, raw	147

The data are for the 75 top scoring foods by category in the analytical database.

## 4 Discussion

Herbs and spices, a relatively understudied category, contain numerous antioxidant micronutrients and are high in carotenoids and flavonoids. Going beyond the nutrition label, the present preliminary NRFa11.3 scoring system gave the highest ratings to herbs and spices closely followed by green leafy vegetables, cocoa powder, and red and orange vegetables. Consistent with past studies (14), anthocyanin-rich berries were the most nutrient-rich fruit.

The present evaluation of nutrient density, based on the NRF model, went beyond fiber, potassium, and vitamin C. Green leafy, red/orange, and cruciferous vegetables are among the main dietary sources of carotenoids, plant-based pigments with reported anti-oxidant properties (8). Cocoa powder, chocolate, wine and tea (especially green tea) are high in total flavonoids (23–25). Both carotenoids and flavonoids are reported to reduce the risk of non-communicable disease, notably CVD and type 2 diabetes (26–30). Both clinical and observational studies have also pointed to beneficial effects of consuming whole berries, juices, and anthocyanin extracts on reducing diabetes and CVD risks (31–33). The benefits of flavonoids, notably flavan-3-ols have been linked to brewed black tea consumption in the US and in France (31–34). Brewed black tea is the main source of flavonoids in the US diet. In France some of the flavonoids may come from wine.

Often treated as condiments or flavor enhancers, herbs and spices contain compounds with well-established antioxidant, anti-inflammatory, and anti-microbial properties (35–39). Most of the reported benefits relate to cardiovascular health (35), though potential benefits to diabetic control, metabolic syndrome (39), stress relief, mood and cognition and digestive health have been noted as well (35, 36). There is extensive literature on the potential health benefits of long term consumption of chili pepper, black pepper, ginger, turmeric, thyme, rosemary, and garlic (36, 37). In a clinical study, saffron and its bioactive components were linked to improved dietary intakes and reduced body weight (40). The present analyses clearly show that herbs and spices contain multiple nutrients and components of interest (35–40).

There are several challenges to capturing nutrient density of herbs and spices. First, databases are still incomplete. Not all herbs and spices in the USDA SR-28 (now SR Legacy) database could be matched with the USDA flavonoid database. In general, the match between SR-28 data and USDA flavonoid data was good for vegetables and fruit but not for herbs and spices. This is a research gap to be addressed, especially since herbs and spices received some of the highest nutrient density scores.

Second, the base of calculation can be 100 g or 100 kcal. The present calculations were based on 100 g as opposed to calories or portion size. Culinary serving sizes for herbs and spices items can be very small (41, 42). The FDA RACC values (Reference Amounts Customarily Consumed) are 0.125 for saffron, 0.2 g for bay leaf and between 1 g and 5 g for most dry herbs (41).

Third, there are no Dietary Reference Intakes for flavonoids at this point and no Recommended Daily Allowances exist. The provisional standard for flavonoids in the NP model was set at 250 mg/d. This value was based on estimates of flavonoid consumption in 2011–16 NHANES that were 205 mg/d for total flavan-3-ols (of which 86 mg/d catechins) (24). Most flavan-3-ols came from brewed black tea. The present standard of 250 mg/d was well below a recent published guideline for flavan-3-ols of 400–600 mg/d (43). Based on a recent review and meta analyses of published data (43), flavan-3-ol intakes in the range of 400–600 mg/d were associated with some degree of cardiometabolic protection, based on moderate evidence from clinical trials where participants were fed very large doses of flavan-3-ols, often in the range of 800–2,000 mg/day (43). Since flavan-3-ols represent about 80% of total flavonoid intakes in the US, that would suggest a potential guideline for total flavonoids to be in the order of 480 to 720 mg/day. Such values are far above the observed patterns of flavonoid consumption in the US where the major source of flavonoids is tea.

The setting of Dietary Reference Intakes is complicated by differential bioavailability of plant based bioactive compounds (44, 45). Carotenoids are fat soluble and are poorly absorbed in a fat free diet (44). Flavonoids are water soluble though availability may be poor. The present values ought to be viewed as only preliminary, since there are no agreed upon DRI standards for flavonoids (43) and their solubility and bioavailability are still in question (44). Work on improving estimates of flavonoid intakes is ongoing (34, 45).

Herbs and spices may have a role in human nutrition that goes beyond replacing sodium chloride in cooked or processed foods (36). The FDA defines spices as substances used for seasoning rather than for any nutritional value. Spices may be exempted from the FDA nutrition labeling requirements if they contain “insignificant” amounts of nutrients that are listed on the Nutrition Facts panel (41). As a result, dried herbs and powdered spices are sometimes relegated to the category of “non-foods.” However, small serving sizes may be compensated for by increased frequency of use. Based on the present results, the contribution of herbs and spices to healthy diets should not be overlooked.

Flavonoids are not currently part of regulatory guidance; however, they can be included in NP algorithms to evaluate the full healthfulness of plant-based foods. Polyphenols and other bioactive compounds are reported to contribute to anti-inflammation (46), anti-aging (47), and therapeutic potential (48). Although the present NP model was based on nutrients and elements with reputed antioxidant activity, it must be noted that it has no connection to the former USDA ORAC (Oxygen Radical Absorbance Capacity) score (49–51).

Developed by the National Institute of Health and Aging (NIH), the ORAC value purported to measure the antioxidant capacity of different foods. The present NP model and does not represent any attempt to calculate “antioxidant power” of foods per serving or any other amount (49–51). The present goal was to show alternative ways to assess the healthfulness of spices, herbs, vegetables and fruits using criteria other than fiber and vitamin C. What is more, the antioxidant value of polyphenols including flavonoids has been questioned (52, 53). Nonetheless, functional ingredients such as carotenoids and flavonoids, that had not been used in NP models before was novel and herbs and spices were the category of most interest. NP methodology needs to innovate to and incorporate the latest databases.

## 5 Conclusion

The present analyses illustrate how NP models can incorporate antioxidant micronutrients and other functional ingredients as new data on flavonoids and polyphenols become available. Incorporating carotenoids and flavonoids into an NP model is relatively new. The present findings point to a very high content of nutrients and functional ingredients in herbs and spices. Normally treated as seasonings, herbs and spices can be an important source key nutrients in the US diet.

## Data availability statement

All databases used for the present calculations are publicly available and can be downloaded from the USDA FoodData Central website https://fdc.nal.usda.gov/.

## Author contributions

AD: Conceptualization, Data curation, Formal analysis, Funding acquisition, Investigation, Methodology, Project administration, Resources, Software, Supervision, Validation, Visualization, Writing – original draft, Writing – review & editing.
