# Supplementary material for: A novel Nutrient Rich Food (NRFa11.3) score uses flavonoids and carotenoids to identify antioxidant-rich spices, herbs, vegetables, and fruit
Source: Front Nutr. 2024 Apr 18;11:1386328. doi: 10.3389/fnut.2024.1386328 (PMC11063353; doi:10.3389/fnut.2024.1386328)
Supplement: Supplementary file 1 [file Table_1.doc]

Supplementary Material

**Supplementary Table 1:** Means and SEM values (per 100g) for protein, fiber, vitamin C, and potassium and energy density (kcal/100g) by food category.

| Food Category | N | Protein g/100g | SEM | Fiber g/100g | SEM | Vitamin C  mg/100g | SEM | Potassium mg/100g | SEM | ED kcal/100g | SEM |
| --- | --- | --- | --- | --- | --- | --- | --- | --- | --- | --- | --- |
| Citrus fruit | 11 | 0.92* | 0.11 | 2.27* | 0.47 | 39.3 | 5.0 | 160.5* | 10.3 | 42.36* | 3.80 |
| Citrus juice | 11 | 0.53* | 0.03 | 0.31* | 0.07 | 33.8 | 2.6 | 148.4* | 9.5 | 35.55* | 3.05 |
| Fruit | 49 | 0.77* | 0.08 | 2.09* | 0.19 | 19.18 | 5.4 | 198.4* | 16.5 | 68.08* | 6.61 |
| Melons | 4 | 0.62* | 0.08 | 0.95* | 0.27 | 30.9 | 11.6 | 197.2* | 33.3 | 35.75* | 2.72 |
| Olives | 3 | 0.95* | 0.06 | 3.00* | 0.25 | 0.80 | 0.4 | 19.7* | 11.2 | 113.7* | 18.5 |
| Berries, raw | 24 | 1.06* | 0.13 | 5.17* | 0.77 | 105.1 | 69.0 | 174.7* | 18.5 | 57.71* | 5.54 |
| Dried fruit | 7 | 4.23* | 1.74 | 6.84* | 1.19 | 8.5 | 6.7 | 637.3* | 121.7 | 294.7 | 12.3 |
| Green leafy veg | 31 | 2.44* | 0.29 | 2.41* | 0.27 | 32.8 | 5.3 | 351.2* | 25.9 | 26.19* | 2.10 |
| Cruciferous veg | 18 | 2.03* | 0.21 | 2.47* | 0.18 | 42.8 | 5.2 | 258.7* | 15.9 | 26.56* | 1.96 |
| Red-orange veg | 24 | 1.16* | 0.08 | 1.92* | 0.20 | 39.6 | 10.8 | 290.5* | 19.5 | 38.67* | 4.34 |
| Other vegetables | 71 | 2.16* | 0.22 | 2.58* | 0.16 | 20.8 | 3.7 | 291.8* | 18.8 | 51.52* | 4.37 |
| Beans | 22 | 11.76 | 2.21 | 7.02* | 1.08 | 7.7 | 1.8 | 633.4* | 127.0 | 172.7* | 31.8 |
| Nuts | 13 | 14.62 | 2.21 | 8.85* | 0.61 | 4.8 | 3.0 | 603.6* | 52.2 | 570.3* | 39.7 |
| Cocoa | 4 | 12.25 | 3.85 | 27.58* | 8.23 | 0.10 | 0.06 | 1393.0 | 413.0 | 267.0 | 43.7 |
| Chocolate | 3 | 10.04 | 2.14 | 7.93* | 4.33 | 0.17 | 0.17 | 611.7 | 132.6 | 572.3* | 34.9 |
| Herbs and spices | 54 | 11.65 | 0.98 | 21.96 | 1.97 | 38.7 | 11.0 | 1322.3 | 136.2 | 266.5 | 17.0 |
| Coffee and tea | 15 | 0.10* | 0.06 | 0.04* | 0.03 | 0.03 | 0.02 | 31.3* | 8.5 | 7.33* | 4.10 |
| Wine | 9 | 0.16* | 0.05 | 0.00* | 0.00 | 0.00 | 0.00 | 74.5* | 13.6 | 87.33* | 10.1 |
| Grains | 7 | 10.83a | 1.39 | 8.67* | 1.95 | 0.00 | 0.00 | 351.6* | 66.1 | 298.4* | 30.9 |
| Cranberry products | 6 | 0.56* | 0.16 | 0.58* | 0.23 | 11.0 | 6.54 | 51.5* | 19.0 | 102.5* | 25.3 |
| Total | 386 | 4.25 | 0.32 | 6.00 | 0.48 | 28.9 | 4.88 | 445.8 | 29.9 | 119.1 | 7.52 |

SEM Standard Error of the Mean. Statistics are one way ANOVA with post hoc Dunnett’s test with herbs and spices as the control group. Herbs and spices are compared to all other groups. Items with * are significantly different from the herbs and spices category. Statistical significance determined at p value < 0.05.

**Supplementary Table 2 :** Means and SEM values for nutrient density subscores for nutrients to encourage (NR4) and nutrients to limit (LIM) and total NRF4.3 nutrient density scores per 100g by food category.

| Food Category | N | NR4 subscore | SEM | LIM subscore | SEM | NRF4.3  score | SEM |
| --- | --- | --- | --- | --- | --- | --- | --- |
| Citrus fruit | 11 | 58.28* | 6.18 | 0.22* | 0.08 | 58.06* | 6.15 |
| Citrus juice | 11 | 43.96* | 2.95 | 0.28* | 0.11 | 43.68* | 3.03 |
| Fruit | 49 | 31.39* | 3.94 | 0.79* | 0.25 | 30.60* | 3.91 |
| Melons | 4 | 44.62* | 14.16 | 0.77 | 0.22 | 43.85* | 14.05 |
| Olives | 3 | 14.06* | 0.67 | 57.69* | 15.1 | -43.63* | 14.64 |
| Berries, raw | 24 | 60.82* | 6.54 | 0.91* | 0.69 | 59.90* | 6.68 |
| Dried fruit | 7 | 58.00* | 12.76 | 14.25 | 11.4 | 43.75* | 19.67 |
| Green leafy veg | 31 | 58.49* | 6.21 | 1.83* | 0.38 | 56.66* | 6.23 |
| Cruciferous veg | 18 | 67.85* | 6.46 | 3.38* | 1.76 | 64.46* | 7.18 |
| Red-orange veg | 24 | 51.40* | 7.59 | 3.74* | 1.23 | 47.66* | 8.14 |
| Other vegetables | 71 | 42.63* | 2.71 | 1.98* | 0.38 | 40.65* | 2.79 |
| Beans | 22 | 75.31* | 10.82 | 4.65* | 1.32 | 70.66* | 11.03 |
| Nuts | 13 | 83.48* | 5.87 | 43.18* | 7.49 | 40.30* | 11.63 |
| Cocoa | 4 | 142.71 | 35.45 | 30.11 | 9.35 | 112.60 | 35.08 |
| Chocolate | 3 | 66.07* | 22.98 | 120.1* | 16.9 | -54.01* | 36.43 |
| Herbs and spices | 54 | 154.95 | 8.77 | 19.31 | 3.66 | 135.64 | 10.16 |
| Coffee and tea | 15 | 1.27* | 0.43 | 0.93* | 0.66 | -0.03* | 0.69 |
| Wine | 9 | 1.74* | 0.45 | 0.17* | 0.06 | 1.52* | 0.46 |
| Grains | 7 | 62.68* | 10.84 | 6.31* | 3.37 | 56.38* | 11.45 |
| Cranberry products | 6 | 16.89* | 6.32 | 12.06 | 7.51 | 4.83* | 11.84 |
| Total | 386 | 63.46 | 2.82 | 7.83 | 0.99 | 55.94* | 2.84 |
|  |  |  |  |  |  |  |  |

SEM Standard Error of the Mean. Statistics are one way ANOVA with post hoc Dunnett’s test with herbs and spices as the control group. Herbs and spices are compared to all other groups. Items with * are significantly different from the herbs and spices category. Statistical significance determined at p value < 0.05.

**Supplemental Table 3:** Means and SEM values for total carotenoid and flavonoids (both expressed as %DV per 100g) by food category. Also shown are %DV for the vitamin and mineral component of the NR11 subscore.

| Food Category | N* | Sum %DV carotenoids | SEM | Sum %DV vitamins and minerals | SEM |  | N | Sum %DV flavonoids | SEM |
| --- | --- | --- | --- | --- | --- | --- | --- | --- | --- |
| Citrus fruit | 11 | 15.96* | 7.37 | 52.24* | 5.01 |  | 11 | 15.40* | 2.41 |
| Citrus juice | 11 | 3.59* | 1.38 | 42.41* | 2.92 |  | 11 | 8.41* | 0.70 |
| Fruit | 49 | 13.94* | 4.13 | 28.06* | 4.01 |  | 44 | 4.82* | 1.27 |
| Melons | 4 | 76.93a | 25.81 | 41.01* | 13.57 |  | 4 | 0.17* | 0.09 |
| Olives | 3 | 26.85a | 0.57 | 41.45* | 0.84 |  | 3 | 1.10* | 0.50 |
| Berries, raw | 24 | 4.99* | 0.86 | 50.41* | 6.06 |  | 20 | 35.08 | 7.97 |
| Dried fruit | 7 | 6.73* | 4.76 | 43.71* | 5.63 |  | 7 | 3.63* | 1.84 |
| Green leafy veg | 31 | 163.43* | 11.17 | 63.07* | 8.01 |  | 31 | 9.84* | 2.89 |
| Cruciferous veg | 18 | 58.92 | 10.66 | 61.44* | 6.48 |  | 18 | 6.63* | 4.65 |
| Red-orange veg | 24 | 115.26 | 14.22 | 53.15* | 7.97 |  | 23 | 1.03* | 0.39 |
| Other vegetables | 71 | 24.76* | 4.76 | 39.16* | 2.74 |  | 70 | 3.75* | 0.88 |
| Beans | 22 | 11.17* | 4.02 | 72.72* | 12.37 |  | 22 | 8.39* | 4.61 |
| Nuts | 13 | 10.51* | 9.71 | 163.90 | 16.14 |  | 12 | 2.62* | 1.19 |
| Cocoa | 4 | 0.67* | 0.23 | 123.58 | 32.55 |  | 4 | 63.77 | 19.66 |
| Chocolate | 3 | 0.50* | 0.28 | 118.75 | 41.34 |  | 3 | 31.61 | 25.43 |
| Herbs and spices | 54 | 87.23 | 17.52 | 159.44 | 9.56 |  | 18 | 40.85 | 9.79 |
| Coffee and tea | 15 | 0.05* | 0.04 | 1.87* | 0.60 |  | 12 | 13.69* | 4.01 |
| Wine | 6 | 0.04* | 0.04 | 2.61* | 0.76 |  | 9 | 20.73* | 8.33 |
| Grains | 7 | 3.45* | 1.38 | 92.48* | 14.73 |  | 7 | 2.33* | 0.83 |
| Cranberry products | 6 | 2.45* | 0.82 | 21.74* | 7.61 |  | 6 | 5.50* | 1.48 |
| Total | 383 | 44.48 | 3.87 | 66.19 | 3.19 |  | 335 | 10.80 | 1.17 |

SEM Standard Error of the Mean. Statistics are one way ANOVA with post hoc Dunnett’s test with herbs and spices as the control group. Herbs and spices are compared to all other groups. Items with * are significantly different from the herbs and spices category. Statistical significance determined at p value < 0.05.

**Supplemental Table 4:** Means and SEM values for components to encourage subscore (NR11), nutrients to limit subscore (LIM) and the final nutrient density score NRFa11.3 by food category.

| Food Category | N* | NR 11 subscore | SEM | LIM subscore | SEM |  | NRF a11.3 | SEM |
| --- | --- | --- | --- | --- | --- | --- | --- | --- |
| Citrus fruit | 11 | 82.16* | 7.69 | 0.22* | 0.08 |  | 81.93* | 7.67 |
| Citrus juice | 11 | 54.09* | 3.31 | 0.28* | 0.11 |  | 53.81* | 3.38 |
| Fruit | 49 | 44.06* | 6.39 | 0.79* | 0.25 |  | 43.26* | 6.37 |
| Melons | 4 | 118.11a | 30.84 | 0.77a | 0.22 |  | 117.34a | 30.92 |
| Olives | 3 | 69.41* | 1.22 | 57.69* | 15.15 |  | 11.72* | 15.95 |
| Berries, raw | 24 | 82.97* | 9.40 | 0.91* | 0.69 |  | 82.06* | 9.52 |
| Dried fruit | 7 | 53.11* | 6.17 | 14.25a | 11.36 |  | 38.86* | 14.75 |
| Green leafy veg | 31 | 209.98a | 18.26 | 1.83* | 0.38 |  | 208.15a | 18.20 |
| Cruciferous veg | 18 | 123.72* | 14.83 | 3.38* | 1.76 |  | 120.33* | 15.40 |
| Red-orange veg | 24 | 164.60* | 13.04 | 3.74* | 1.23 |  | 160.86a | 13.19 |
| Other vegetables | 71 | 63.44* | 5.11 | 1.98* | 0.38 |  | 61.46* | 5.13 |
| Beans | 22 | 89.24* | 13.85 | 4.65* | 1.32 |  | 84.58* | 14.05 |
| Nuts | 13 | 174.40a | 17.89 | 43.18* | 7.49 |  | 131.22* | 20.79 |
| Cocoa | 4 | 188.02a | 43.35 | 30.11a | 9.35 |  | 157.91a | 42.77 |
| Chocolate | 3 | 150.86a | 66.96 | 120.09* | 16.86 |  | 30.77* | 76.92 |
| Herbs and spices | 54 | 226.36 | 18.62 | 19.31 | 3.66 |  | 207.05 | 18.84 |
| Coffee and tea | 15 | 12.87* | 3.34 | 0.93* | 0.66 |  | 12.22* | 3.65 |
| Wine | 6 | 22.50* | 8.42 | 0.17* | 0.06 |  | 24.92* | 9.09 |
| Grains | 7 | 96.79* | 15.52 | 6.31a | 3.37 |  | 90.48* | 15.57 |
| Cranberry products | 6 | 29.69* | 8.37 | 12.06a | 7.51 |  | 17.63* | 14.31 |
| Total | 383 | 110.89 | 5.11 | 7.83 | 0.99 |  | 103.60 | 5.02 |

SEM Standard Error of the Mean. Statistics are one way ANOVA with post hoc Dunnett’s test with herbs and spices as the control group. Herbs and spices are compared to all other groups. Items with * are significantly different from the herbs and spices category. Statistical significance determined at p value < 0.05.
